# *Nostoc sphaeroids* Kütz powder ameliorates diet-induced hyperlipidemia in C57BL/6j mice

**DOI:** 10.29219/fnr.v63.3618

**Published:** 2019-12-25

**Authors:** Fenfen Wei, Yinlu Liu, Cuicui Bi, Bo Zhang

**Affiliations:** Research Institute for Science and Technology of Functional Foods; Beijing Key Laboratory of Bioactive Substances and Functional Foods, Beijing Union University, Beijing, China

**Keywords:** Nostoc sphaeroids Kütz, high-fat diet, lipid, metabolism disorders, mice

## Abstract

**Background:**

Hypercholesterolemia is a disease associated with numerous health problems. Growing evidence indicates that hypercholesterolemia, hyperlipidemia is closely linked to chronic inflammation, which can lead to cardiovascular disease, fatty liver disease, and type 2 diabetes.

**Objective:**

The purpose of this study was to investigate the protective effect of Nostoc sphaeroids Kütz (NO) on diet-induced hyperlipidemia in mice.

**Design:**

At first, experimental animals received a high-fat diet (HFD) for 4 weeks, and then received a HFD supplemented with 2.5% or 7.5% NO for 6 weeks. In the current study, results show that treatment with NO decreases weight gain and liver index induced by HFD. In addition, the serum levels of TC, TG and LDL are significantly decreased in NO treatment groups.

**Results:**

From the results of Oil Red staining and Hematoxylin and eosin staining (HE), treatment with NO significantly reduces liver lipid accumulation and protect liver structure. Further analysis reveals that NO has positive effects on liver lipid metabolism and inflammation, as showed by the lower protein expressions of FAS and SREBP-1, the lower concentrations of TNF-α, IL-1β, IL-6, and the lower gene expressions of TNF-α, IL-1β, IL-6 and NF-kB.

**Conclusions:**

Our results indicate that NO may significantly ameliorate diet-induced hyperlipidemia, which is possibly associated with improving liver lipid metabolism and reducing chronic inflammation.

## Popular scientific summary

- *Nostoc sphaeroids* Kütz improved lipid metabolism disorders in mice induced by a high-fat diet.
- The beneficial effects of *Nostoc sphaeroids* Kütz were mediated through the regulation of inflammation and modulation of hepatic FAS and SREBP-1 protein expression.

Hyperlipidemia is associated with numerous health problems and reduced life expectancy (1). Growing evidence indicates that hyperlipidemia is closely linked with chronic inflammation, which can lead to cardiovascular disease, fatty liver disease, and type 2 diabetes (2, 3). Previous studies have indicated that pro-inflammatory cytokines are closely associated with chronic diseases such as tumor necrosis factor-α (TNF-α), interleukin-1β (IL-1β), and interleukin-6 (IL-6) (4). High-fat or high-sugar diets are associated with an increased prevalence of atherosclerosis, which can lead to coronary artery disease (5, 6). While the causes of hyperlipidemia are complex, unbalanced diets have been postulated as a major cause of energy metabolism disruption and excessive lipid accumulation in multiple organs, especially in the liver (7–9). The liver plays a key role in lipid metabolism (10); therefore, a reduction in fat intake and lipid accumulation could be effective ways to ameliorate hyperlipidemia and associated metabolic disorders. Drugs that are currently available for treating hyperlipidemia, such as statins and fibrates, have shown very limited effectiveness, have side effects, and are expensive (11). Conversely, many researchers have focused on developing substances with lipid-lowering functions from natural products. The habitual consumption of foods with therapeutic effects on metabolic disorders may be an effective and manageable way to prevent hyperlipidemia.

Blue-green algae (BGA), one of the most primitive life forms on earth, have been utilized for food and as medicine in Asian countries for several thousands of years. Species such as *Nostoc flagelliforme* Born. et Flah, *Spirulina platensis*, and *Nostoc sphaeroides* Kütz (NO) contain a wide range of bioactive compounds and have various functions (12, 13). One of the most intriguing BGA species is NO, also known as Ge-Xian-Mi in China, which has been used to promote health for centuries (14). According to previous studies, NO biomass is high in polysaccharides, amino acids, protein, vitamins, and minerals (15). They also have bioactive properties, such as antiviral, antitumor, antidiabetic, anti-inflammatory, and lipid-modulating effects (15, 16). Previous studies have shown that NO and its extracts significantly reduced plasma total cholesterol (TC) and triglyceride (TG) levels in male C57BL/6j mice and may have the potential to reduce the risk of atherosclerosis (16–18). Nevertheless, it remains unknown whether NO could have any effect on body weight or regulatory effects on liver lipid accumulation. Therefore, it is important to investigate the lipid-lowering mechanisms and anti-inflammatory functions of NO as a food supplement and to explore its potential applications in the food industry.

In the present study, we aimed to explore whether NO supplementation affects serum lipid levels, liver lipid accumulation, pro-inflammatory cytokines, and lipid biosynthesis proteins during a high-fat diet (HFD).

## Materials and methods

### Chemicals and reagents

The NO used in this study was provided by Hunan Yandi Bioengineering Co., Ltd. (Hunan, China). Dry NO was crushed into powder form at −20°C for 2 h, then used directly in this study. The main components of the NO used in the present research were fiber (47.3%), protein (30.8%), moisture (5.6%), and ash (5.7%); the remaining components were vitamins and minerals; components were identified by Societe Generale de Surveillance S.A. (Shanghai, China). Assay kits for total cholesterol (TC), triglyceride (TG), high-density lipoprotein (HDL), and low-density lipoprotein (LDL) were acquired from Nanjing Jiancheng Bioengineering Institute (Nanjing, China). ELISA kits for TNF-α, IL-1β, and IL-6 were purchased from Wuhan Huamei Biological Engineering co. LTD (Wuhan, China). Total RNA extraction regent, SYBR Green Master primer, and oligo (dT)18 were obtained from Roche (Basel, Switzerland). Oil Red O and hematoxylin were purchased from Wuhan servicebio Co., Ltd. (Wuhan, China). Antibodies against fatty acid synthase (FAS) and sterol regulatory element-binding protein-1 (SREBP-1) were acquired from Wuhan servicebio Co., Ltd. (Wuhan, China).

### Animals and diets

Forty C57BL/6j male mice were purchased from Beijing Vital River Laboratory Animal Technology Co., Ltd. (Beijing, China) at 6 weeks of age, and were allowed to acclimate to their surroundings for 1 week. All mice were housed in an air-conditioned room (temperature 22 ± 2°C), relative humidity of 50–60%, 12 h light/dark cycle).

### Experimental protocols

At the end of adaptive feeding for 1 week, mice were randomly divided into four groups (*n* = 10 in each group, one animal per cage): the control group, the HFD group, the NO (2.5%) group, and the NO (7.5%) group. Control group animals received an AIN-93M control diet, while other groups were fed a modified HFD based on AIN-93M for 4 weeks. During weeks 5–10, control group animals were maintained on a control diet, the model group was fed an HFD, the low-dose group was fed an HFD supplemented with 2.5% NO (w/w, 2.5% NO), and the high-dose group was fed with HFD supplemented with 7.5% NO (w/w, 7.5% NO). All feed was provided by Beijing Keao Xieli Feed Co., Ltd., (Beijing, China). Dietary compositions are shown in Table 1. The total kilocalories of HFD, NO (2.5%), and NO (7.5%) are 5,150, 5,140, and 5,214 kcal, respectively, and there is no significant difference in dietary calories between HFD-fed groups. At week 4, blood samples were collected by retro-orbital bleeding and transferred into centrifuge tubes. Serum samples were obtained by centrifugation at 4°C and 4,000 r/min for 10 min, and then stored at −80°C for further analysis. At the end of the experiment, mice were fasted for 16 h. The following day, animals were weighed and anesthetized by intraperitoneal injection of barbital at 9:00 a.m. Blood samples were collected from the sock vein. Liver tissue was collected and stored at −80°C for quantitative real-time reverse-transcription (qRT–PCR) and Western blot analysis, or fixed in paraformaldehyde for histological analysis. All animal procedures were in accordance with the Animal Care and Use Committee of Beijing Union University.

**Table 1:** Composition of assay diets

Ingredient (g)	Control diet	High-fat diet	*Nostoc sphaeroids* Kütz (NO) (2.5%)	NO (7.5%)
Cornstarch	465.7	235.7	233.2	228.2
Casein	140	110	110	110
Dextrinized cornstarch	155	155	155	155
Sucrose	100	100	100	100
Soybean oil	40	40	40	40
Choline bitartrate	2.5	2.5	2.5	2.5
Fiber^a^	50	50	50	50
Mineral mix^b^	35	35	35	35
Vitamin mix^c^	10	10	10	10
L-Cysteine	1.8	1.8	1.8	1.8
Lard	0	150	150	150
Cholesterol	0	10	10	10
Yolk	0	100	100	100
NO powder	0	0	25	75
Total	1,000	1,000	1022.5	1067.5

a Solka-Floc cellulose

b AIN-93 mineral mix

c AIN-93 vitamin mix.

### Biochemical analysis in serum and hepatic tissue

The lipids in serum and liver were determined as described previously (19). Serum concentrations of TC, TG, HDL, and LDL were determined using commercial assay kits (Nanjing Jiancheng Bioengineering Institute, China). The liver inflammatory cytokine concentrations of TNF-α, IL-1β, and IL-6 were determined using CUSABIO ELISA kits. All assays were performed according to the manufacturer’s instructions.

### Quantitative real-time reverse-transcription PCR

Total RNA from mice liver was extracted using a total RNA extraction kit (Servicebio, China) according to the manufacturer’s protocol. Two micrograms of total RNA samples were used to synthesize cDNA using the RevertAid First Strand cDNA Synthesis Kit (Thermo Scientific, USA). Quantitative real-time reverse-transcription PCR (qRT–PCR) was performed in triplicate using SYBR Green and a Light Cycler 480 Real-Time PCR System (Roche Diagnostics). Each well was loaded with a 20 μL sample, containing 2.5 μL cDNA, 2.0 μL target primers, 8.0 μL water, and 12.5 μL Kapa SYBR Fast Master Mix. Hot-start PCR was performed for 40 cycles. Each cycle consisted of denaturation for 15 sec at 95°C, annealing for 30 sec, and elongation for 30 sec at 60°C. Roche Light Cycler software (version 1.5.0, Roche Diagnostics) was utilized for data analysis. The results were analyzed using the 2^−ΔΔCt^ method of analysis. Mean expression levels for control group mice were set as 100%. The primers used are shown in Table 2.

**Table 2:** Primer pairs used for the real-time quantitative reverse-transcription (PCR) analysis

GenBank ID	Gene name		Primer sequence (5' to 3')
NM_007393.3	β-actin	Forward	GTGACGTTGACATCCGTAAAGA
		Reverse	GTAACAGTCCGCCTAGAAGCAC
NM_009045.4	NF-kB	Forward	AAGCACAGATACCACCAAGACAC
		Reverse	CGCACTGCATTCAAGTCATAGTC
NM_001278601.1	TNF-α	Forward	GCATCCAGCTTCAAATCTCGC
		Reverse	TGTTCATCTCGGAGCCTGTAGTG
NM_008361.4	IL-1β	Forward	CCCTCACACTCACAAACCACC
		Reverse	CTTTGAGATCCATGCCGTTG
NM_031168.2	IL-6	Forward	CCCCAATTTCCAATGCTCTCC
		Reverse	CGCACTAGGTTTGCCGAGTA

### Western blotting

One hundred milligrams of liver tissue per sample were homogenized in a commercial total protein extraction solution (Servicebio, Wuhan, China). Total protein lysates were fractionated by 10% SDS-PAGE (Servicebio, Wuhan, China) and electro-blotted onto a Polyvinylidene Fluoride membrane (Millipore, Massachusetts, USA). The primary antibodies used in this assay were FAS (1:1,000, 37 KD), SREBP-1(1:1,000, 57 KD), and β-Actin (1:1,000, 40 KD), which were purchased from Servicebio (Wuhan, China). Membranes were blocked with 5% non-fat milk for 1 h in Tris-Buffered Saline Tween buffer and probed with primary antibodies overnight at 4°C. Then, membranes were incubated with horseradish peroxidase-conjugated secondary antibody. Protein bands were visualized using chemiluminescence reagent (Millipore, Massachusetts, USA). The band values were calculated using AlphaEase FC software (Alpha Innotech).

### Oil Red O staining

Mice liver tissue was prepared into frozen sections, which were stained with Oil Red O (Servicebio, China) for 10 min, and then counter-stained with hematoxylin for 5 min. The sections were then analyzed under light microscopy (Olympus, Japan). The qualification for lipid accumulation was analyzed using ImageJ software (Microsoft Windows).

### Hematoxylin and eosin staining

Mice liver was fixed in 4% paraformaldehyde solution and made into paraffin sections. Paraffin sections (4 μm) were cut and then stained with hematoxylin and eosin (Servicebio, China). The sections were then analyzed under light microscopy (Olympus, Japan).

### Statistical analysis

Statistical analysis was conducted using SPSS software for windows (version 22). Data were assessed using one-way ANOVA and Newman–Keuls pair-wise comparison. *P*-values < 0.05 were considered significant differences. All data from these assays are shown as mean ± SEM.

## Results

### NO ameliorates HFD-induced obesity and fat accumulation in mice

**Fig. 1:**
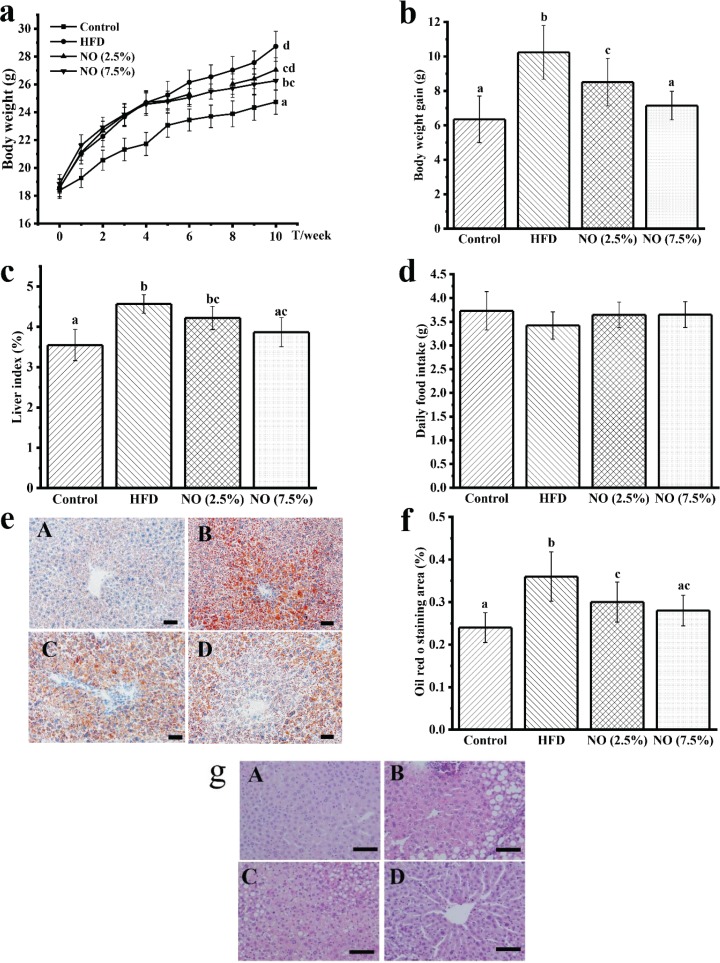
NO reduces body weight and lipid accumulation in HFD-fed mice. Effects of NO on (a) body weight; (b) body weight gain; (c) liver index, liver index (%) = $\frac{Liver\;weight\;\left(g\right)}{Body\;weight\;\left(g\right)}*$ 100%; (d) daily food intake; (e) Oil Red O staining, 200×; (f) Lipid area ratio, and Oil Red O staining ration = $\frac{Lipid\;area\;\;}{Total\;are\;}*$ 100%; (g) HE staining, 400×. Scale bar = 100 μm, A: Control, B: HFD, C: NO (2.5%), D: NO (7.5%). Bars marked with different letters represent statistically significant (*P* < 0.05), whereas bars labeled with the same letter indicate no statistically significant difference between the groups (*P* > 0.05). Values represent mean ± SEM; *n* = 10 in each group.

Animal weight and food intake were monitored on a weekly basis. After 10 weeks, HFD-fed mice showed a significant increase in body weight (Fig. 1a), while mice on NO (7.5%) diet for 6 weeks had significantly lower body weight compared with the HFD group mice (Fig. 1a). There was a significant increase in body weight in the HFD group, while supplementing with NO decreased the body weight (Fig. 1b). After 10 weeks, we observed a significant increase in the liver index of the HFD-fed mice compared with the control feeding group mice, while the NO (7.5%) group had significantly lower liver index (Fig. 1c). Lipid accumulation visualizations are shown in Fig. 1e–f. Liver tissue images revealed that lipids accumulated greatly in HFD group animals, while the NO (7.5%) supplementation group had clearly a lesser amount of lipid accumulation (Fig. 1e). The results of quantification of lipid accumulation are shown in Fig. 1f. The HFD group mice showed the highest levels of lipid accumulation, while treatment with NO significantly reduced the lipid accumulation (Fig. 1f). There was severe liver damage in the HFD group and there were a lot of fat vacuoles compared with control group animals (Fig. 1g); however, supplementation with NO (7.5%) was associated with a more complete liver structure than the control group. Based on the results in Fig. 1d, all groups showed no significant differences in daily food intake, suggesting that the effects of NO on body weight and liver were not due to lower food consumption.

### NO ameliorates serum lipid levels induced by HFD

The serum lipid levels, including TC, TG, HDL, and LDL, were investigated in this study. After 4 weeks, the levels of serum lipid in HFD-fed groups increased significantly, indicating that hyperlipidemia model animals were successfully generated. After 6 weeks of NO treatment, the levels of TC, TG, and LDL were significantly lower and the level of HDL was significantly higher, especially in the NO (7.5%) group (Table 3), compared with the HFD group.

**Table 3:** Effects of high-fat diet (HFD) on lipid levels in serum

Group	Control	HFD	2.5% *Nostoc sphaeroides* Kütz (NO)	7.5% NO
Total cholesterol (TC) (mmol/L)	3.12 ± 0.64^a^	8.29 ± 0.61^b^	8.49 ± 0.91^b^	8.62 ± 0.84^b^
Triglyceride (TG) (mmol/L)	1.01 ± 0.23^a^	1.87 ± 0.37^b^	1.89 ± 0.43^b^	1.97 ± 0.43^b^
High-density lipoprotein (HDL) (mmol/L)	1.87 ± 0.41^a^	1.45 ± 0.30^b^	1.41 ± 0.31^b^	1.14 ± 0.43^b^
Low-density lipoprotein (LDL) (mmol/L)	0.51 ± 0.092	1.08 ± 0.17^b^	1.10 ± 0.17^b^	1.08 ± 0.11^b^

Values represent mean ± SEM; *n* = 10 in each group. Superscript different letters between groups represent statistically significant differences (*P* < 0.05). Instances of the same letter between groups indicate that no statistically significant difference was found between the groups (*P* > 0.05).

### NO decreases inflammation in mice induced by HFD

**Fig. 2:**
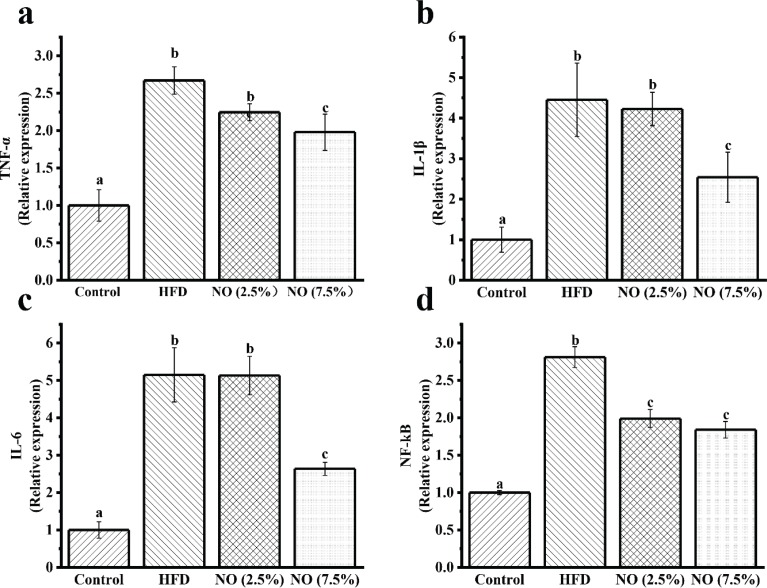
Analysis of pro-inflammatory cytokines in mice liver tissue. Relative mRNA expression levels of (a) TNF-α, (b) IL-1β, (c) IL-6, and (d) NF-kB in liver tissue were assessed using qRT-PCR. Values represent mean ± SEM, *n* = 6 in each group. Letters represent statistically significant differences (*P <* 0.05). Instances of the same letter between groups indicate that no statistically significant difference was found between the groups (*P* > 0.05).

Previous studies have suggested that HFDs are linked to elevated levels of pro-inflammatory cytokines, such as TNF-α, IL-1β, and IL-6 (20). Concentrations of pro-inflammatory cytokines and messenger mRNA expression levels in liver tissue were measured (Table 5). The nuclear factor-kappa B (NF-kB) pathway regulates the production of pro-inflammatory cytokines in target tissues and leads to chronic inflammation in HFD-fed mice (21). Therefore, the NF-kB mRNA expression was measured. Concentrations of TNF-α, IL-1β, and IL-6 were higher in HFD-fed mice compared with those of the control diet mice, while these cytokines were significantly less, and were affected in a dose-dependent manner, in the NO treatment groups (Table 5). The mRNA expression levels of TNF-α, IL-1β, IL-6, and NF-kB in the HFD-fed mice were significantly increased, while cytokine expression levels were altered in a dose-dependent manner with NO treatment. NO treatment resulted in expression levels closer to that of the control diet mice than the HFD-fed mice with increasing NO concentrations (Fig. 2). These results indicate that NO has a positive effect on the inflammation induced by an HFD.

### NO downregulates protein expression in mice liver

**Fig. 3:**
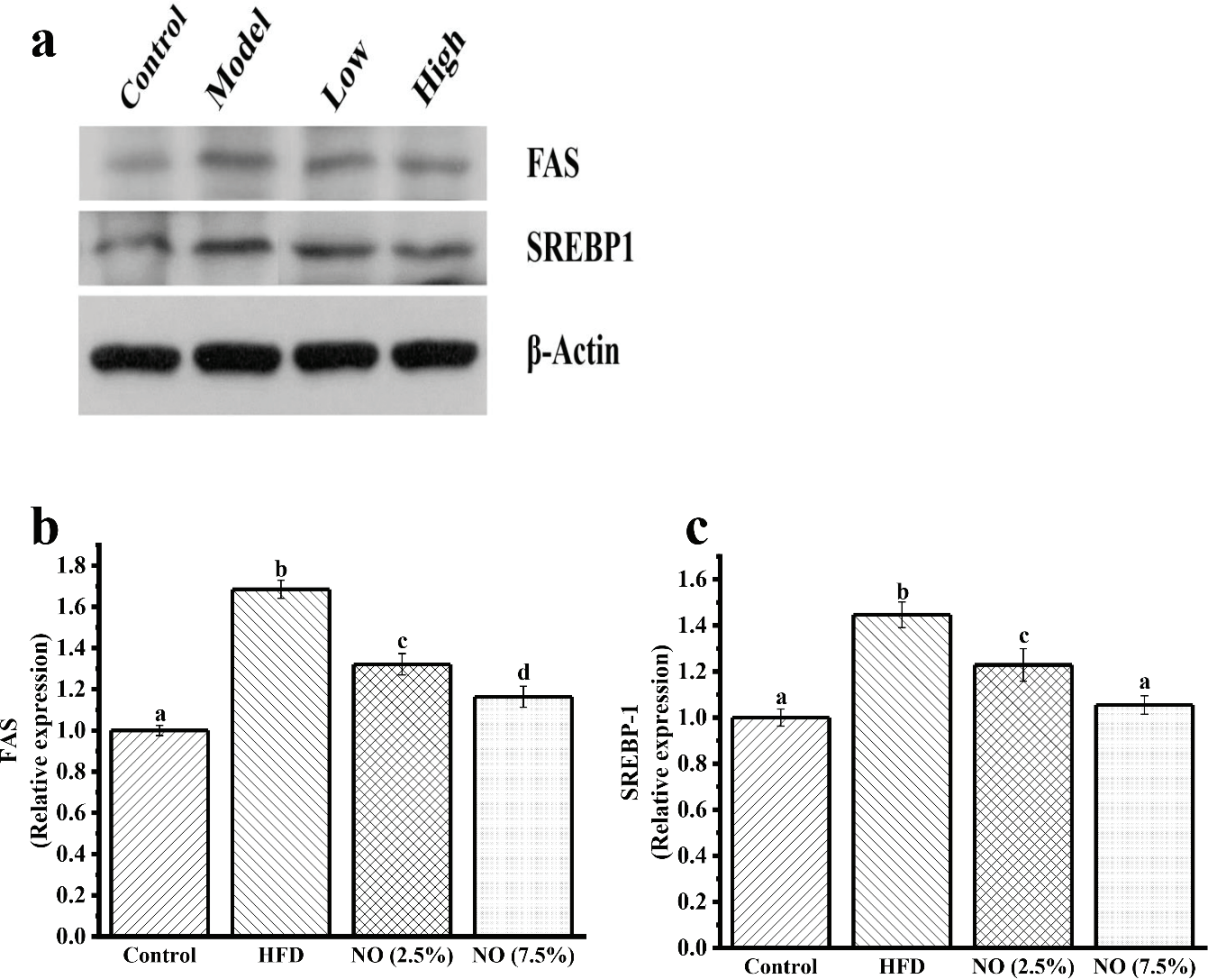
Effects of NO on FAS and SREBP-1 protein expression in mice liver. Model: HFD, Low: NO (2.5%), High: NO (7.5%). Representative immunoblots for FAS and SREBP-1 proteins are shown in **a**. Relative expression levels normalized to internal control (β-Actin) are shown in **b** and **c**. Data represent the mean ± SEM, *n* = 6. Letters represent statistically significant differences (*P <* 0.05). Instances of the same letter between the groups indicate that no statistically significant difference was found between the groups (*P* > 0.05).

Studies have shown that the expression of proteins involved in lipid biosynthesis, such as FAS and SREBP-1, are elevated in the liver tissue of the HFD-fed mice (22,23). Hence, FAS and SREBP-1 protein expression levels in the liver tissue were examined. Our results showed that the mice supplemented with NO had a significantly lower expression of FAS and SREBP-1 protein in liver tissue than the HFD group mice (Fig. 3).

## Discussion

Dietary intervention has been a major strategy in combating global hyperlipidemia. In spite of the various health-promoting properties associated with NO treatment, there has been a limited understanding of the underlying mechanisms of action. As hyperlipidemia and chronic inflammation are closely related, in the present study, we studied lipid profile in serum, inflammation, and lipid metabolism in liver. Our results indicate that NO had beneficial effects on hyperlipidemia in mice induced by HFD.

Initially, mice fed with an HFD for 4 weeks exhibited dyslipidemia and hypercholesterolemia, as shown by significantly greater serum TC, TG, HDL, and LDL levels compared with the control group. These results are consistent with previous studies that showed that HFD-fed animals developed dyslipidemia (24, 25), indicating that hyperlipidemia model mice were successful established. Then, after 4 weeks of HFD, the mice received an HFD supplemented with NO (2.5%), NO (7.5%), or without NO for 6 weeks.

This study shows that supplementing diet with NO significantly reduces TC, TG, and LDL, and increases HDL in serum (Table 4), which is consistent with a previous study finding that NO treatment significantly lowered plasma lipid (18). Although the serum lipid levels have not reached the control levels, it still shows that NO exerts a positive effect towards ameliorating lipids and has the potential to prevent hyperlipidemia in mice. The liver is the main organ for lipid metabolism, and liver lipid homeostasis is controlled by many mediators and enzymes (26, 27). SREBP-1 and FAS are supposed to play an important role in the process of lipid metabolism (28, 29). Therefore, we investigated the expression of these mediator proteins in liver tissue by Western blotting. The results of the present study suggest that NO consumption suppresses SREBP-1 and FAS protein expression in a dose-dependent manner. Our results indicate that lipid metabolic disorders could be modulated by dietary intervention, and NO can play a role by modulating SREBP-1 and FAS protein expression in the liver of HFD-fed mice. In other words, it is reasonable to conclude that NO ameliorates serum lipids probably by decreasing lipids synthesis in the liver. Previous studies also indicate that when the expression of SREBP-1 and FAS was inhibited, the synthesis of fat and lipid will be affected (30, 31). Research among SREBP-1 and FAS inhibitors is also very popular (32). Also, NO-supplemented mice showed a significant reduction in lipid droplets from Oil Red O staining, and based on HE staining results, treatment with NO was associated with a more complete liver structure. Overall, from the pathology results, we could conclude that NO may protect the liver structure in the HFD-fed mice. Therefore, it is reasonable to believe that NO inhibited the synthesis of lipid in the liver, reducing the burden of liver lipid metabolism, thereby reducing lipid droplets in the NO groups.

**Table 4:** Effects of *Nostoc sphaeroides* Kütz (NO) on serum lipid levels of high-fat diet (HFD) mice

Group	Control	HFD	2.5% NO	7.5% NO
Total cholesterol (TC) (mmol/L)	3.96 ± 0.74^a^	7.65 ± 1.06^b^	5.93 ± 1.07^c^	5.77 ± 0.91^c^
Triglyceride (TG) (mmol/L)	0.71 ± 0.16^a^	1.81 ± 0.39^b^	1.2 ± 0.38^c^	1.03 ± 0.32^ac^
High-density lipoprotein (HDL) (mmol/L)	1.85 ± 0.39^a^	1.36 ± 0.35^b^	1.63 ± 0.29^c^	1.69 ± 0.25^c^
Low-density lipoprotein (LDL) (mmol/L)	0.63 ± 0.18^a^	1.24 ± 0.28^b^	1.09 ± 0.14^b^	0.98 ± 0.12^c^

Values represent mean ± SEM; *n* = 10 in each group. Superscript different letters between groups represent statistically significant differences (*P* < 0.05). Instances of the same letter between groups indicate that no statistically significant difference was found between the groups (*P* > 0.05).

In addition, the HFD-fed mice treated with NO showed a significantly lower gene expression level (TNF-α, IL-1β, IL-6, and NF-kB) than those not treated with NO (Fig. 2). Also, the ELISA assay results are in accordance with RT-PCR (Table 5). Our results indicate that NO could reverse chronic inflammation induced by HFD to a significant extent. Previous studies have demonstrated that NO markedly reduced mRNA expressions of pro-inflammatory cytokines, including NF-α, IL-1β, and IL-6 in mice (16, 17). Chronic inflammation has been reported to increase under hypercholesterolemic conditions (33) and is the mechanism through which hypercholesterolemia induces tissue damage, resulting in the over-production of pro-inflammatory cytokines such as TNF-α, IL-1β, and IL-6 in the HFD-fed mice (34, 35). In the present study, HFD in mice for 10 weeks resulted in chronic inflammation. When the diet was supplemented with NO for 6 weeks, the chronic inflammation in liver was reduced and the serum lipids were ameliorated. Therefore, it can be concluded that NO could reduce inflammation and finally ameliorate the serum lipids in the HFD-fed mice.

**Table 5:** Effects of pro-inflammatory cytokine levels in liver tissue

Group	Control	High-fat diet	*Nostoc sphaeroides* Kütz (NO) (2.5%)	NO (7.5%)
TNF-α (pg/mL)	174.03 ± 20.47^a^	334.09 ± 32.66^b^	290.97 ± 29.95^c^	205.26 ± 26.05^d^
IL-1β (pg/mL)	147.27 ± 27.75^a^	327.46 ± 39.53^b^	302.88 ± 36.63b	218.16 ± 29.63^c^
IL-6 (pg/mL)	16.07 ± 4.13^a^	34.21 ± 4.26^b^	27.34 ± 4.67c	19.79 ± 3.61^a^

Values represent mean ± SEM, *n* = 10 in each group. Superscript letters represent statistically significant differences (*P <* 0.05). Instances of the same letter between groups indicate that no statistically significant difference was found between the groups (*P* > 0.05).

## Conclusions

In conclusion, our results have shown that NO has beneficial effects for the HFD-fed mice. NO decreased the levels of TC, TG, LDL in serum, and ameliorated inflammation in the HFD-fed mice. The beneficial effects were primarily attributed to the suppression of FAS and SREBP-1 protein expression, and the inhibition of TNF-α, IL-1β, IL-6, and NF-kB gene expression.
